# Cardiovascular Entropy and Mortality Prediction in Hemodialysis Patients

**DOI:** 10.3390/jcm15093244

**Published:** 2026-04-24

**Authors:** Longin Niemczyk, Katarzyna Romejko, Katarzyna Buszko, Daniel Schneditz, Stanisław Niemczyk

**Affiliations:** 1 Department of Nephrology, Dialysis and Internal Medicine, Medical University of Warsaw, 1a Banacha Street, 02-097 Warsaw, Poland; 2 Department of Internal Diseases, Nephrology and Dialysis, Military Institute of Medicine—National Research Institute, 128 Szaserów Street, 04-141 Warsaw, Poland; kromejko@wim.mil.pl (K.R.); sniemczyk@wim.mil.pl (S.N.); 3 Department of Biostatistics and Biomedical Systems Theory, Ludwik Rydygier Collegium Medicum, Nicolaus Copernicus University in Toruń, Ul. Jagiellońska 15, 87-067 Bydgoszcz, Poland; buszko@cm.umk.pl; 4 Otto Loewi Research Center, Division of Physiology, Medical University of Graz, Neue Stiftingtalstrasse 6/V, 8010 Graz, Austria

**Keywords:** amplitude-aware permutation entropy, cardiovascular signals, chronic kidney disease, mortality

## Abstract

**Background/Objectives**: The main cause of death in patients with chronic kidney disease (CKD) is of cardiovascular origin. Entropy-based analysis of physiological signals reflects system irregularity, complexity, and adaptive capacity. Amplitude-aware permutation entropy (AAPE) is a signal analysis method suitable for assessing complex cardiovascular dynamics, and growing evidence suggests that measures of physiological signal variability and complexity may have prognostic value. This study aimed to evaluate whether AAPE can predict mortality in CKD patients undergoing hemodialysis (HD), with and without diabetes. The aim of this study was to assess whether AAPE analysis of cardiovascular signals following the administration of a glucose bolus directly into the extracorporeal circuit during hemodialysis (HD)—a method originally used to treat intradialytic hypotension and to study the kinetics of glucose, insulin, and C-peptide in patients with and without type 2 diabetes—can predict mortality in patients with chronic kidney disease (CKD) undergoing hemodialysis (HD), both with and without diabetes. **Methods**: After seven years of follow-up, mortality outcomes were analyzed in relation to AAPE-derived parameters. **Results**: Higher mortality was associated with smaller differences in AAPE of mean arterial pressure (MAP) and diastolic arterial pressure (DIA) before and after intravenous glucose administration (*p* = 0.009 and *p* = 0.016, respectively). Higher tonicity was associated with higher survival (*p* = 0.01). Additionally, greater reductions in AAPE of systolic arterial pressure (SYS) and larger differences in AAPE of ejection time (EJT) and total peripheral resistance (TPR) were associated with increased mortality. **Conclusions**: These findings suggest that entropy analysis reflects cardiovascular adaptability and may serve as a prognostic biomarker in HD patients.

## 1. Introduction

Health is based on a stable equilibrium state regulated by numerous complex control mechanisms. However, to maintain body homeostasis, there are numerous and apparently random regulatory activities providing sufficient degrees of freedom to cope with complex perturbations. In the state of disease, organisms often lose the ability to regulate physiological processes, which is usually associated with a simplification of the control system. Currently, new methods based on nonlinear analyses for studying the complexity of regulatory processes are emerging for application in clinical medicine. One approach to assess the complexity of physiological systems is based on the measurement of entropy.

The term ‘entropy’ comes from the Greek word ‘entropía’, meaning ‘transformation’ and ‘change’. First introduced by Rudolf Clausius in 1864, the concept of entropy was developed to explain the spontaneity and direction of physical and chemical processes. This term is also a key notion in information theory. In medicine, entropy is used to measure disorder, irregularity, complexity, unpredictability and information. The state of low entropy results from regular or periodic values of various quantities, and such an equilibrium state is predictable for future periods of time, e.g., seconds, minutes, or hours. In this context, entropy is of interest in biological and medical sciences. Pathological states are usually characterized by reduced entropy. There are various methods to measure entropy with a variety of potential application areas, including medical sciences [1]. One of the methods is the measurement of amplitude-aware permutation entropy (AAPE), a variant of permutation entropy (PE) [2].

Cardiovascular comorbidities such as hypertension, atherosclerosis, heart failure, coronary and peripheral artery disease, and the derangement of the autonomic nervous system, including baroreceptor dysfunction or impaired vasopressin response, are common findings in chronic kidney disease (CKD). They intensify with the decline in kidney function, are most severe in patients treated with dialysis, and are the main cause of morbidity and mortality in CKD [3,4]. AAPE is one of many signal-processing methods used to analyze complex cardiovascular data. It appears that AAPE could also be applied to identify features and specific complexities of cardiovascular signals that may be associated with a particular health condition or disease risk, also in patients with CKD which constitute a major, global health burden [5,6,7].

In our previous study, AAPE analysis of cardiovascular signals revealed impaired autonomic regulation in patients with end-stage renal disease (ESRD) treated with hemodialysis (HD), both with and without type-2 diabetes mellitus, in response to dialysis and intravenous glucose administration. After glucose injection, AAPE responses differed between both groups: the amplitude-aware permutation entropy of heart rate (AAPE(HR)) and ejection time (AAPE(EJT)) increased significantly only in patients with diabetes, whereas blood pressure-related entropy indices including amplitude-aware permutation entropy of systolic (AAPE(SYS)), diastolic (AAPE(DIA)), mean arterial pressure (AAPE(MAP)), and total peripheral resistance (AAPE(TPR)) increased predominantly in non-diabetic participants. Amplitude-aware permutation entropy of cardiac output (AAPE(CO)) increased after glucose administration in both groups, while the amplitude-aware permutation entropy of stroke volume (AAPE(SV)) remained unchanged. These findings indicate differential autonomic and hemodynamic responses to metabolic perturbation between diabetic and non-diabetic patients [8].

We subsequently examined whether changes in signal complexity during HD could predict mortality in the studied cohort. There is growing evidence that measures of physiological signal variability and complexity may have prognostic value in predicting mortality and long-term clinical outcomes. A reduction in heart rate variability (HRV) has been found to be an independent predictor of mortality in CKD patients treated with HD [9,10]. Additionally, heart rhythm complexity appears to be a promising tool for estimating long-term cardiovascular outcomes in peritoneal dialysis patients [11]. Additionally, the analysis of cardiovascular signals variability using entropy measurements may help to assess the life expectancy of patients with diseases other than decreased kidney function or diabetes [12]. Therefore, the measurement of cardiovascular entropy could be useful for predicting survival in patients with certain conditions, including diabetes and end-stage renal disease.

The aim of this study was to determine whether the change in AAPE of hemodynamic variables was associated with 7-years mortality in CKD patients undergoing HD, both with and without diabetes.

## 2. Materials and Methods

This report is the continuation of the study conducted in the Department of Internal Diseases, Nephrology and Dialysis, Military Institute of Medicine—National Research Institute in Warsaw, Poland between 2016 and 2018 [8]. The study population comprised 31 CKD patients treated with hemodialysis, of which 20 were without (NDM) and 11 with type-2 diabetes mellitus (DM), the latter being treated either with a diabetic diet only or additionally with insulin. The median dialysis vintage was 2 years. The study included patients aged over 18 years who had undergone HD treatment for at least three months using a peripheral arteriovenous fistula with an access flow rate of over 600 mL/min and a minimum extracorporeal blood flow rate of 250 mL/min. The exclusion criteria were clinical signs of infection, symptomatic anemia, hormonal therapy, and oral diabetogenic drugs (glucocorticoids, thiazide diuretics, beta-blockers, immunosuppressants such as tacrolimus and cyclosporine A, psychiatric and neurological medications such as atypical antipsychotics, statins, nicotinic acid, and thyroid hormones) for patients with diabetes [8]. We also excluded patients in severe clinical condition, with neoplastic processes, short life expectancy, or frailty. All patients signed an informed consent to participate in the study. The study protocol was accepted by the local ethics committee, the Internal Review Board (IRB) of the Military Institute of Medicine—National Research Institute in Warsaw according to local regulations and recommendations of the Helsinki Declaration on research involving human subjects and approved under IRB approval number KB53/WIM/2012, dated 17 October 2012, with amendments obtained on 20 March 2013 and 18 January 2017, and with the extension of approval obtained on 18 January 2017.

Patients were studied during regular HD. Participants were asked not to eat or drink for at least three hours prior to the start of HD. Insulin therapy was discontinued for 8 h before starting the study in DM patients. A supine body position was maintained throughout the test. Thirty minutes after the beginning of dialysis, a 40% glucose solution was given at a rate of 0.5 g/kg dry weight (a constant rate of 1 mL/s) and cardiovascular signals were observed for 60 min after the start of the infusion. Heart rate and arterial blood pressures were noninvasively and continuously recorded with the use of an inflatable cuff mounted on the index or middle finger of the contralateral access arm using the Portapres^®^ system (Finapres Medical Systems, Enschede, The Netherlands) with a sampling frequency of 100 Hz. Signals such as heart rate (HR), systolic blood pressure (SYS), diastolic blood pressure (DIA), mean arterial pressure (MAP), total peripheral resistance (TPR), cardiac output (CO), stroke volume (SV), and ejection time (EJT) were extracted. For each signal, AAPE was calculated at the start of HD, before and after the glucose injection, and at the end of the observation phase [8].

Mortality was assessed after seven years of follow-up, and the potential for mortality prediction based on AAPE results was examined.

Quantitative data are presented as means and standard deviations (SD), whereas categorical data are expressed as numbers and percentages. The Shapiro–Wilk test was used to assess the distribution of data. In the absence of a normal distribution, non-parametric tests were applied. Comparisons between groups were made using t-tests for independent variables or Mann–Whitney tests. Survival analysis was performed, with survival curves being compared using the Log-rank test. Receiver Operating Characteristic (ROC) analysis was used to evaluate mortality prediction based on AAPE changes. A mixed-effects model with random effects was constructed to assess factors influencing changes in AAPE. Statistical analysis was performed at a significance level of α = 0.05.

## 3. Results

**Figure 1:**
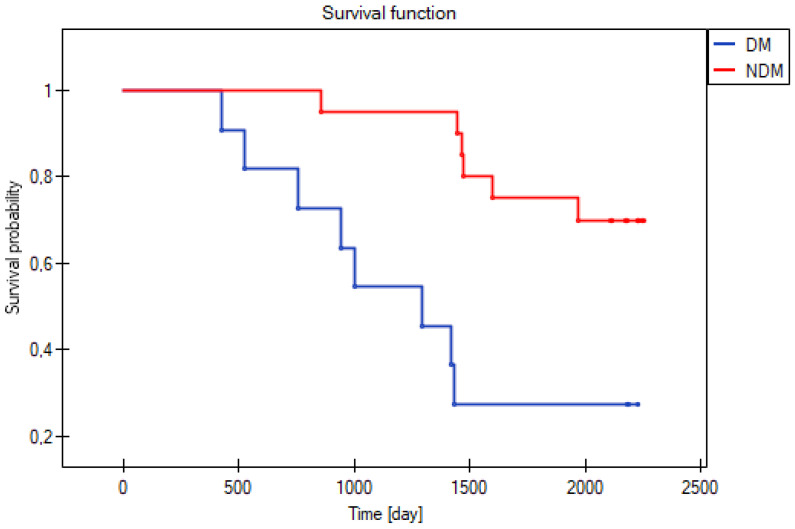
Kaplan–Meier survival probability for patients with (DM, blue line) and without (NDM, red line) diabetes mellitus.

Among the 31 patients studied in 2018, 20 were individuals without diabetes mellitus (NDM) (65% men) and 11 were patients with diabetes mellitus (DM) (54.5% men). Fourteen patients passed away during the follow-up period, six NDM patients (30%) and eight DM individuals (73%) (Figure 1, Table 1). The majority of fatalities (88%) was of cardiovascular origin: 8 patients died from chronic heart failure [4 DM and 4 NDM], 3 from sudden cardiac arrest of undetermined cause [2 DM and 1 NDM], 1 from hemorrhagic stroke [1 DM], while 2 patients died from cancer [1 DM and 1NDM].

**Figure 2:**
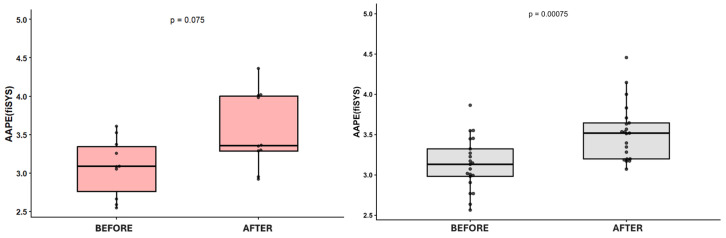
Amplitude-aware permutation entropy of systolic blood pressure after glucose administration in participants with (DM, red) and without (NDM, gray) diabetes mellitus. Dots show individual data.

**Figure 3:**
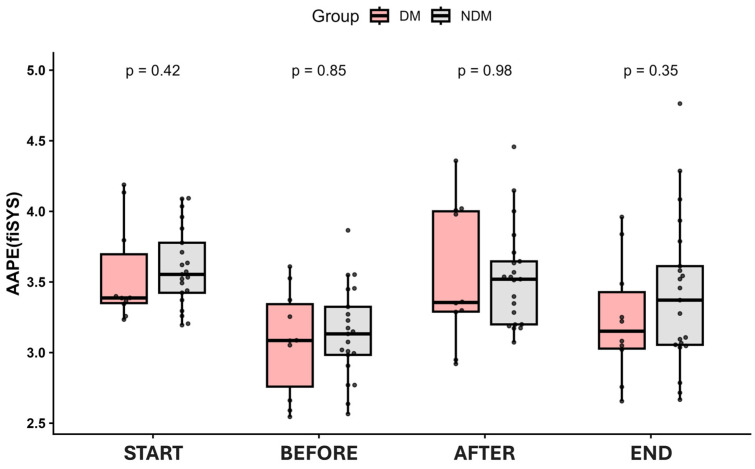
Amplitude-aware permutation entropy of systolic blood pressure in participants with (DM, red) and without (NDM, gray) diabetes mellitus. *p*, probability to reject the null-hypothesis (DM = NDM). Dots show individual data.

Among various entropy parameters examined, only AAPE(SYS) reduction served as a criterion for differentiating between NDM and DM patients. After glucose administration, AAPE(SYS) rose in both NDM and DM patients. Moreover, its increase was statistically significant in NDM individuals only (*p* < 0.001). There was only a trend for AAPE(SYS) to increase in DM patients (*p* = 0.075) (Figure 2). This increase was followed by a decline reaching almost the same values as before the intervention in DM patients, contrary to the NDM group, where the AAPE(SYS) at the end of the intervention was higher than before the glucose administration. Therefore, in patients with DM, the decline of AAPE(SYS) and its return to baseline values after glucose administration were more pronounced than in NDM participants (Figure 3).

We also found that AAPE(SYS) changed with HD duration (time) and with ultrafiltration volume (UFV) (*p* = 0.089, R^2^ = 0.2 CI (0.1–0.34)) (Table 2).

**Figure 4:**
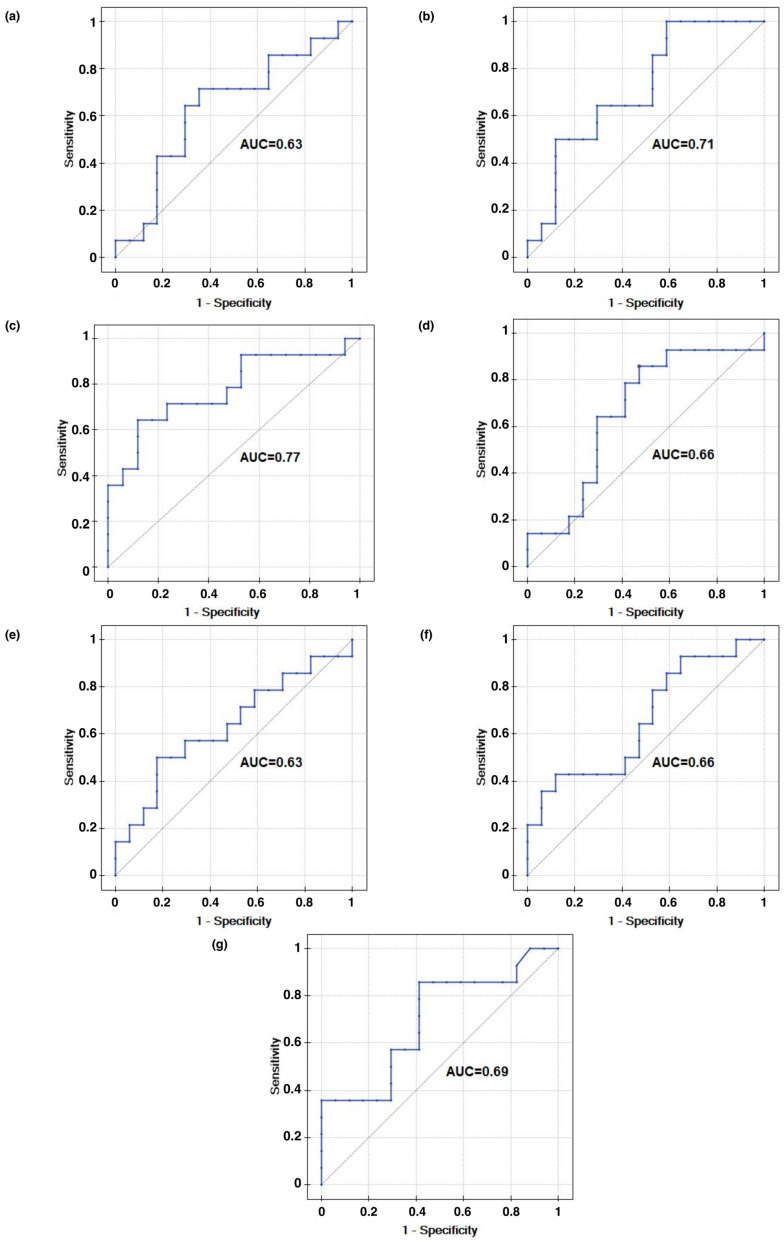
Receiver operating characteristics for amplitude-aware permutation entropy reduction as a marker for stratifying mortality: (**a**) dAAPE(SYS), (**b**) dAAPE(MAP), (**c**) dAAPE(DIA), (**d**) dAAPE(EJT), (**e**) dAAPE(TPR), (**f**) tonicity, (**g**) AAPE(SYS).

According to ROC analysis (Figure 4a), higher mortality was associated with a stronger reduction in AAPE(SYS) after glucose administration. The absolute change in entropies caused by the intervention was therefore also examined for other hemodynamic variables. Mortality was also associated with the change in AAPE(MAP) (dAAPE(MAP)). Increased dAAPE(MAP) was associated with better outcomes (*p* = 0.009) (Table 3, Figure 4b). Additionally, lower mortality was associated with higher difference in entropy for diastolic blood pressure (dAAPE(DIA)) (*p* = 0.016) (Table 3, Figure 4c). Greater changes in AAPE(EJT) and AAPE(TPR) (dAAPE(EJT), dAAPE(TPR)) tended to be associated with higher mortality, although these differences were not significant (*p* = 0.339 and *p* = 0.256, respectively; Table 3, Figure 4d,e). Higher tonicity was also correlated with higher survival rate (*p* = 0.01) (Figure 4f). Increased mortality was also associated with diabetes (*p* = 0.002).

A power analysis was conducted to compare changes in AAPE(SYS) values before and after the intervention. At an assumed significance level of α = 0.05, the power of Student’s t-test was 0.99.

## 4. Discussion

In our study, changes in entropy-derived hemodynamic signals during HD and the response to intravenous glucose administration were associated with mortality, mostly of cardiovascular origin. Specifically, a stronger reduction in AAPE(SYS) following glucose administration and higher values of dAAPE(EJT) and dAAPE(TPR) were related to increased mortality. In contrast, higher dAAPE(MAP) and a greater change in entropy for AAPE(DIA) were associated with improved survival. Higher tonicity, reflected in more adaptive entropy responses, also correlated with higher survival rates.

Entropy in physiological signals reflects irregularity, complexity, and adaptability. Associations between cardiovascular signal entropy and mortality have been observed in the general population. Data from The Irish Longitudinal Study on Aging (TILDA) demonstrated that higher entropy of blood pressure signals predicted all-cause mortality in older adults, supporting the notion that entropy-based measures reflect general cardiovascular adaptability and prognostic risk [13]. Some previous studies also showed that entropy has prognostic value in patients with CKD, including those undergoing dialysis. Heart rhythm complexity, measured by multiscale entropy, is often reduced in ESRD. In HD patients, heart rhythm complexity was found to be significantly lower in non-survivors compared with survivors, suggesting that heart rate multiscale entropy could serve as a useful predictor of mortality [10]. Studies in peritoneal dialysis patients also showed that reduced heart rhythm complexity, as assessed by multiscale entropy, was associated with higher cardiovascular mortality. Lower entropy values likely reflect impaired autonomic regulation, and these findings suggest that measures of heart rhythm complexity may provide independent prognostic information for adverse cardiovascular outcomes in patients with ESRD [11].

In our previous study, we examined the hemodynamic response to glucose administration during HD, one of the methods to treat ultrafiltration-induced hypotension avoiding additional sodium load. The action of hypertonic is based on osmotic vasopressin release and intravascular volume expansion [14,15,16]. The administration of glucose during HD is safe even in diabetic patients because excess glucose not deposited in insulin-dependent tissue is entirely removed by extracorporeal clearance during HD within a short period of time [17,18]. Glucose infusions during HD prevent symptomatic IDH and do not cause hypertensive episodes [16,19]. We also observed that entropy of cardiovascular signals decreased during dialysis but partially rebounded after glucose infusion. This rebound suggested a partial restoration of autonomic control in response to metabolic stress. Responses varied by signal type and by patients’ metabolic status, distinguishing diabetic from non-diabetic individuals [8]. In the current study, a larger drop in AAPE(SYS) after glucose administration may indicate an inadequate or maladaptive autonomic response. These patients might have impaired baroreflex function or altered sympathetic–parasympathetic balance, which could contribute to their higher mortality. Similar mechanisms were suggested in critically ill populations, where greater metabolic instability may contribute to endothelial dysfunction and oxidative stress [20].

Blood pressure control during hemodialysis is critical for cardiovascular stability. In our study, higher dAAPE(MAP) and larger dAAPE(DIA) after glucose administration were associated with better survival, suggesting that patients with adjustable blood pressure regulation can better tolerate the hemodynamic stress of dialysis. Conversely, higher dAAPE(TPR) and dAAPE(EJT) were linked to increased mortality, indicating impaired vascular responsiveness. These findings are consistent with previous studies showing that dynamic measures of blood pressure, such as intradialytic variability, are stronger predictors of cardiovascular events in HD patients than static blood pressure values. Therefore, entropy-based indices may provide additional insight into cardiovascular regulation and risk [21].

Previous studies in HD patients highlighted the prognostic value of dynamic blood pressure measurements. A systematic review and meta-analysis demonstrated that greater systolic blood pressure variability was associated with higher risks of all-cause and cardiovascular mortality in HD patients, indicating that variability in blood pressure over time reflects cardiovascular instability and poor outcomes [22]. Similarly, a large observational study showed that increased intradialytic systolic blood pressure variability was independently associated with greater all-cause and cardiovascular mortality, underlining the importance of dynamic rather than static blood pressure dynamics in risk stratification [23]. Entropy-based complexity measurements were also empirically linked to blood pressure dynamics in ESRD, with lower entropy correlating with higher systolic and mean blood pressures, suggesting impaired autonomic control [24]. For example, the study by Yamanaka et al., which included 27 patients treated with HD, found that entropy of HRV was negatively correlated with systolic and mean arterial pressures [25]. Our findings extend these observations by showing that entropy-derived hemodynamic signals, including AAPE(SYS), AAPE(DIA), AAPE(MAP), AAPE(TPR), and AAPE(EJT), are associated with mortality, suggesting that measures of blood pressure complexity may provide additional prognostic information beyond standard variability parameters. Our findings highlight that changes in entropy during HD and after glucose administration may indicate how well patients cope with hemodynamic stress. Those who exhibit larger reductions in AAPE(SYS), higher dAAPE(EJT) and dAAPE(TPR), or smaller increases in dAAPE(MAP) and dAAPE(DIA) may benefit from intensified monitoring and individualized interventions aimed at improving autonomic and hemodynamic stability. The association of hemodynamic-derived entropy measures with mortality is especially relevant because of the high prevalence of cardiovascular disease deaths in this study in particular and in the dialysis population in general. Last, but not least, the controlled administration of hypertonic glucose offers the possibility to assess the diabetic status of patients during HD [26].

While our study offers new insights into the prognostic value of entropy dynamics, it has some limitations. The relatively small, single-center cohort limits generalizability. The exact mechanisms linking entropy changes to factors like autonomic dysfunction or vascular stiffness remain unclear. Future studies should confirm these findings in larger populations and investigate whether entropy-based monitoring can guide interventions to improve outcomes.

## 5. Conclusions

In conclusion, changes in entropy-derived hemodynamic signals during HD in response to intravenous glucose administration were associated with patient survival. A greater reduction in systolic entropy AAPE(SYS) and larger changes in entropies of ejection time dAAPE(EJT) and peripheral resistance dAAPE(TPR) were related to higher mortality, whereas larger changes in diastolic entropy dAAPE(DIA) and mean arterial pressure entropy dAAPE(MAP) were associated with better outcomes. These findings suggest that entropy analysis reflects the adaptive capacity of the cardiovascular system and may serve as a marker of cardiovascular adaptability. Incorporating entropy-based measures into clinical monitoring may provide additional information for risk stratification and should be further explored in dialysis patients.

## Figures and Tables

**Table 1:** Characteristics of the study population.

	Total *n* = 31	NDM *n* = 20	DM *n* = 11	*p*
Sex	Males *n* = 19 Females *n* = 12	Males *n* = 13 Females *n* = 7	Males *n* = 6 Females *n* = 5	0.57
Age, mean ± SD [years]		56.9 ±13.8	69.5 ± 8.0	0.03 **
BMI [kg/m^2^]		27.5 ± 5.0	29.8 ± 4.8	0.17 **
Death	14 (45%)	6 (30%)	8 (73%)	0.022 *

Patients with (DM) and without (NDM) diabetes mellitus; * χ^2^-test; ** Mann–Whitney test; statistically significant *p*-values are in bold type.

**Table 2:** Mixed model with random effects for the difference in amplitude-aware permutation entropy of systolic blood pressure before and after intravenous glucose administration (dAAPE(SYS)), R^2^ = 0.26 95% CI (0.430; 0.195).

	Std. Error	t-Value	*p*-Value
Intercept	0.129	21.515	**<0.001**
time: start vs. after	0.046	−0.861	0.391
time: start vs. before	0.046	−6.347	**<0.001**
time: start vs. end	0.046	−3.506	**0.001**
UFV [mL]	0.048	1.762	0.089

UFV, Ultrafiltration volume; *p*-values < 0.05 are marked in bold.

**Table 3:** Cox proportional hazards regression.

Variable	Chi-Square Statistic	*p*-Value	AIC-Akaike Criterion	R^2^ (Nagelkerke)	Direction of Diagnostic Variable	AUC	*p*-Value
dAAPE7(MAP)	6.893	**0.009**	84.281	0.389	destimulant	0.77	**0.009**
dAAPE7(DIA)	5.779	**0.016**	85.395	0.339	destimulant	0.71	**0.043**
dAAPE7(SYS)	3.264	0.070	87.909	0.208	destimulant	0.66	0.121
dAAPE7(EJT)	0.712	0.399	90.461	0.050	destimulant	0.63	0.204
dAAPE7(TPR)	1.287	0.256	89.887	0.088	stimulant	0.63	0.204
AAPE7(SYS)	2.452	0.117	88.722	0.170	stimulant	0.66	0.130
Tonicity	6.538	**0.010**	84.635	0.374	stimulant	0.70	0.060

dAAPE7(MAP), difference in amplitude-aware permutation entropy of mean arterial pressure before and after the intervention; dAAPE7(DIA), difference in amplitude-aware permutation entropy of diastolic arterial pressure before and after the intervention; dAAPE7(SYS), difference in amplitude-aware permutation entropy of systolic arterial pressure before and after the intervention; dAAPE7(EJT), difference in amplitude-aware permutation entropy of ejection time before and after the intervention; dAAPE7(TPR), difference in amplitude-aware permutation entropy of total peripheral resistance before and after the intervention; AAPE7(SYS), amplitude-aware permutation entropy of systolic arterial pressure; 7 indicates the number of amplitude levels used in the calculation; AUC, area under the curve; *p*-values < 0.05 are marked in bold.

## Data Availability

Original data can be requested from the corresponding author due to ethical regulations and patient privacy considerations.

## Abbreviations

The following abbreviations are used in this manuscript:

AAPE	Amplitude-aware permutation entropy
AAPE(CO)	Amplitude-aware permutation entropy of cardiac output
AAPE(DIA)	Amplitude-aware permutation entropy of diastolic blood pressure
AAPE(EJT)	Amplitude-aware permutation entropy of ejection time
AAPE(HR)	Amplitude-aware permutation entropy of heart rate
AAPE(MAP)	Amplitude-aware permutation entropy of mean blood pressure
AAPE(SV)	Amplitude-aware permutation entropy of stroke volume
AAPE(SYS)	Amplitude-aware permutation entropy of systolic blood pressure
AAPE(TPR)	Amplitude-aware permutation entropy of total peripheral resistance
AUC	Area under the curve
BMI	Body mass index
CKD	Chronic kidney disease
CO	Cardiac output
DIA	Diastolic blood pressure
DM	With diabetes mellitus
EJT	Ejection time
ESRD	End-stage renal disease
HD	Hemodialysis
HR	Heart rate
HRV	Heart rate variability
IRB	Internal Review Board
MAP	Mean arterial pressure
NDM	Without diabetes mellitus
PE	Permutation entropy
ROC	Receiver Operating Characteristic
SV	Stroke volume
SYS	Systolic arterial pressure
TILDA	The Irish Longitudinal Study on Ageing
TPR	Total peripheral resistance
UFV	Ultrafiltration volume
